# Neuronal correlates of label facilitated tactile perception

**DOI:** 10.1038/s41598-018-37877-w

**Published:** 2019-02-07

**Authors:** Timo Torsten Schmidt, Tally McCormick Miller, Felix Blankenburg, Friedemann Pulvermüller

**Affiliations:** 1 Neurocomputation and Neuroimaging Unit (NNU), Department of Education and Psychology, Freie Universität Berlin, 14195 Berlin, Germany; 2 Brain Language Laboratory, Department of Philosophy and Humanities, WE4, Freie Universität Berlin, 14195 Berlin, Germany; 3 Berlin School of Mind and Brain, Humboldt Universität zu Berlin, 10099 Berlin, Germany

## Abstract

It is a long-standing question in neurolinguistics, to what extent language can have a causal effect on perception. A recent behavioural study reported that participants improved their discrimination ability of Braille-like tactile stimuli after one week of implicit association training with language stimuli being co-presented redundantly with the tactile stimuli. In that experiment subjects were exposed twice a day for 1 h to the joint presentation of tactile stimuli presented to the fingertip and auditorily presented pseudowords. Their discrimination ability improved only for those tactile stimuli that were consistently paired with pseudowords, but not for those that were discordantly paired with different pseudowords. Thereby, a causal effect of verbal labels on tactile perception has been demonstrated under controlled laboratory conditions. This raises the question as to what the neuronal mechanisms underlying this implicit learning effect are. Here, we present fMRI data collected before and after the aforementioned behavioral learning to test for changes in brain connectivity as the underlying mechanism of the observed behavioral effects. The comparison of pre- and post-training revealed a language-driven increase in connectivity strength between auditory and secondary somatosensory cortex and the hippocampus as an association-learning related region.

## Introduction

The human brain has the remarkable ability to store knowledge of facts, objects, people, actions, and perceptions, and relate these representations to somewhat arbitrary linguistic labels. The acquisition of new words happens with high speed and efficiency and has been tested in numerous behavioural studies^1–3^. In this context it has been hotly debated as to how far the association of words to particular percepts also leads to changes in perception as such. This question has been addressed in the study of *linguistic relativity*, where multiple observational field studies give strong support of this proposition^4–12^. Within an experimental (i.e. laboratory) context, the *language perception causality (LaPeC)* statement has been formulated as the hypothesis that language can causally affect perceptual discrimination^13^.

Multisensory integration arising from simultaneous input via more than one modality (e.g. visual, auditory, tactile) can influence our perception^14^; at the neural mechanistic level, it may lead to the formation of distributed cell assemblies that form the basis of perceptual discrimination and language comprehension^15^. In a recent behavioural study we reported a paradigm in which implicit associations of pseudowords with tactile stimuli had a causal effect on tactile stimulus discrimination^13^. In the reported study, we applied an association learning paradigm where participants were repeatedly co-presented with language-like auditory input (pseudowords) and tactile stimuli, giving rise to multisensory integration. Half of the tactile stimuli were concordantly (CON) paired with the same redundant pseudowords, while the other half was discordantly (DIS) paired with differing pseudowords. Participants were exposed to these pairings during an intensive training phase twice daily for 1 hour. The participants were not aware of the pairings and as the tactile stimuli were very hard to discriminate (initial d’ of approximately 1); any kind of association between tactile stimuli and pseudwords was formed implicitly. Note furthermore that the pseudowords were entirely irrelevant for the tactile discrimination task applied. After one week of training, subjects had improved their discrimination ability for tactile stimuli, crucially, only for the CON condition but not in the DIS condition, despite equal exposure to all stimuli. Thereby, the first direct evidence for the *LaPeC* statement from a within-subject laboratory experiment was provided.

We have hypothesized that the reported behavioural effects distinguishing the matched con- and discordant conditions are due to increases in neuronal coupling of auditory and somatosensory regions, resulting from Hebbian learning, and we therefore tested this hypothesis using fMRI data which was acquired before and after the training. This is motivated by work on language processing which has demonstrated that words used to speak about actions and perceptions activate different cortical areas, also including sensorimotor cortices, where specific features of the words’ semantic meaning become manifest^16–19^. It has further been demonstrated that auditory and somatosensory activity interact already in early sensory cortices^20^. Such early multisensory integration and interaction between hierarchically low sensory cortices could interlink the neuronal correlates of linguistic representations of spoken word forms, specific acoustic-phonetic sensory features of word forms, or both, with sensorimotor information, thereby giving rise to the *LaPeC* effect. It has been suggested that cell assemblies distributed across language-related as well as motor and sensory areas are the result of Hebbian learning mechanisms (synaptic strengthening by co-activation and synaptic weakening by uncorrelated activation); related predictions have been tested in neurocomputational modelling studies^21^ and in experimental work of language processing^19,22^. Crucially, none of these studies thus far could demonstrate in a controlled laboratory experiment a direct influence on perceptual abilities as such, e.g. a language induced benefit in discrimination abilities. Consecutively, it can be suggested based on this pre-existing work that causal effects of language on perceptual processes exist, and are mediated by interactions of auditory/language circuits with perceptual regions. However, until now, direct experimental tests of this hypothesis had been lacking, as no laboratory experimental paradigms were available that demonstrated effects of cross-modal associations altering perception along with the emergence of functional interactions between sensorimotor and auditory areas relevant to language processing during verbal learning.

To test this hypothesis, we here present fMRI data collected alongside the behavioural learning experiment reported in Miller *et al*.^13^, where every participant underwent fMRI scanning before and after the learning phase. This fMRI study had been designed to test for changes in coupling between auditory and somatosensory cortices. Therefore, during fMRI scanning, participants were presented unimodally with either pseudowords or tactile stimuli. This allowed us to test whether the correlation between pseudowords and co-presented tactile percepts drives the formation of neuronal circuitries of sensory regions from both modalities, following the suggestions of Hebbian learning mechanisms. In addition, the hippocampus with its well-documented contribution to association learning via consolidation and replay to entrain associative mechanisms has been included in our analysis.

We first tested if association learning would lead to a co-activation of somatsosensory perceptual regions during the presentation of associated pseudowords and vice versa. Furthermore, we used the Psychophysiological Interaction (PPI) approach^23^ to quantify task modulated connectivity changes from before to after the training phase. The applied experimental design allowed testing for differences in functional connectivity between pseudowords that were concordantly and discordantly paired with tactile stimuli. We hypothesized that, due to Hebbian learning effects resulting from the implicit learning procedure, coupling between auditory and somatosensory regions would increase. We therefore tested for connectivity changes from the left primary somatosensory (SI) as well as bilateral primary auditory (AI) cortices as seed-regions and hypothesized to find connectivity changes between these regions and further somatosensory (SII) and learning related regions (hippocampus).

## Methods

### Participants

Sixteen healthy, right handed (mean laterality quotient 80.5 ± 16.6 s.d. according to the Edinburgh Handedness Inventory^24^), native German-speaking participants (nine female) took part in this study. Participants provided written informed consent prior to partaking in the study and were compensated for their time. Procedures were approved by the Ethics Committee of the Charité Universitätsmedizin, Campus Benjamin Franklin, Berlin, Germany. All procedure was consistent with the guideline included in the “Declaration of Helsinki – Ethical Principles for Medical Research Involving Human Subjects”. Age ranged from 18 to 34 (mean age 25.2 ± 4.6 SD). All participants were screened for calloused fingers before participation. One participant was excluded due to finger injuries sustained on the last day prior to arriving at the testing facility, leaving N = 15 for all reported analyses.

### Study Design

**Figure 1:**
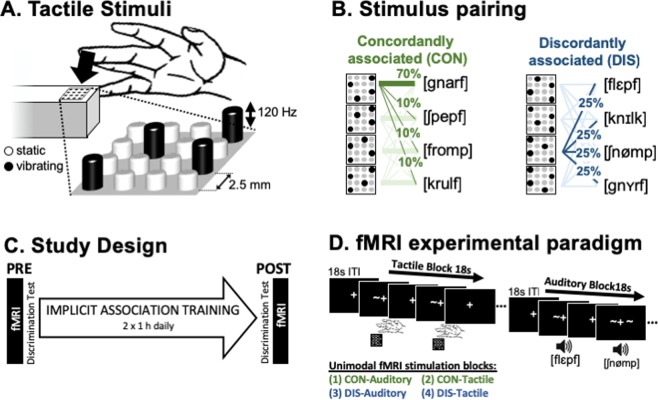
Stimuli and Design. (**A**) Vibrotactile-stimuli were presented on a 4 × 4-pin Braille-like piezoelectric display to the right middle finger. Each stimulus consisted of static (non-vibrating) pins and four pins that vibrated with sigmoidal 120 Hz. (**B**) Tactile-stimuli were equally similar within each of two sets, and because the sets were parity inverse, stimulus comparisons within both sets were equally difficult. In parallel, two sets of four pseudowords were constructed, where pseudowords conformed to German phonological rules. For the implicit learning phase, pairs of tactile-stimuli and pseudowords were defined to form a concordantly associated (CON) and a discordantly associated (DIS) condition. (**C**) During the learning phase, participants were jointly presented with pseudowords and tactile-stimuli to implicitly form associations within the CON condition. fMRI scanning and a discrimination task were conducted before and after the implicit association phase. (**D**) The fMRI paradigm comprised six runs of a unimodal block-design. Each of the four 18 s stimulation-blocks was repeated three times per run (18 s inter-block interval). Each block was comprised of 12 stimuli (each stimulus three times) of 600 ms length intermitted by 900 ms inter-stimulus intervals. Order of stimuli within a block and order of blocks were randomized. A and B reprinted from Miller *et al*.^13^.

Participants were investigated with fMRI before and after the implicit association learning phase of one week (See Fig. 1C) of the original behavioural study, whose results we reported separately in Miller, *et al*.^13^. Details on the training procedure are described with the behavioural dataset. In brief, during training vibrotactile patterned stimuli (Fig. 1A) were either concordantly (CON) or discordantly (DIS) paired with pseudowords to allow for the formation of implicit associations between the verbal stimuli and the tactile stimuli. Participants underwent one-hour training sessions every morning and every afternoon from Monday until Friday morning, where auditory and tactile stimuli were jointly presented. Stimulus assignments were randomized across subjects. No explicit information about the pairings or variations in their consistencies was given. Before and after the learning phase, participants performed a discrimination task to determine if the association of labels affected their tactile stimulus perception. Calculation of d’ (signal detection theory) before and after the training phase demonstrated that training improved the discrimination ability of subjects only for tactile stimuli from the CON condition but not from the DIS condition, and that only in the CON conditions, subjects became capable of discriminating tactile stimuli above chance. For experimental details on the randomization scheme, the discrimination task and the training procedure, please see Miller *et al*. ^13^.

For the fMRI experiment, the same two sets of vibrotactile pattern stimuli and verbal labels were used as in the behavioural study. In short: patterned stimuli were presented on a fMRI-compatible 16-pin Braille-like display (4 × 4 matrix with 2.5 mm spacing) controlled by a programmable stimulator (Piezostimulator, QuaeroSys, St. Johann, Germany). Patterns were composed of four pins vibrating as 120 Hz sinusoidal, while the remaining 12 pins remained static (Fig. 1A). Each set was composed of four patterns of pin-combinations, designed to be minimally different within each set and maximally different between the two sets. Within a tactile-set, each pattern differed from all other patterns by two pin positions. Between the two sets there was no overlap in the patterns. The second set has been created by inversing the first set, therefore stimuli were matched for difficulty to be distinguished from one another. Verbal label stimuli were applied as eight pseudowords, which were maximally different, following the Consonant-Vowel pattern CCVCC (find pseudowords listed in Fig. 1B). All pseudowords conformed to phonetic rules of German. Each of two sets contained four pseudowords, which did not overlap by more than one non-vocalic phoneme in the same position. All pseudowords contain no neighbours, and the bigram and trigram frequencies were matched between sets. All pseudowords were recorded by a male German native speaker. Both vibrotactile and auditory stimuli lasted 600 ms in their presentation.

All participants had equal exposure to all pseudowords and tactile patterns throughout the original behavioural study and the fMRI study at hand. As subjects were not aware of the DIS or CON conditions throughout the experiment, they did not report to have noticed any systematicity in the presentation upon explicit request after the experiment.

### fMRI paradigm and data acquisition

The same experimental procedure was carried out in the PRE and in the POST session (Fig. 1C). In a block design, participants were presented with unimodal stimuli of either tactile patterned stimuli delivered to the right middle finger or verbal auditory stimuli presented via MR-compatible headphones. Each stimulation block belonged to one of four experimental conditions: (1) CON-Auditory (2) DIS-Auditory (3) CON-Tactile (4) DIS-Tactile. Each 18 s block was comprised of the four stimuli from the corresponding condition, each repeated three times in randomized order. The 600 ms stimuli were separated by 900 ms inter-stimulus-intervals. On a screen, a visual fixation cross and ‘~’-cues, marking the stimulation, were presented to motivate the participants to keep their eyes open, as instructed.

To ensure subjects’ attention towards the stimuli, they had to detect outlier-stimuli, for which the pitch (auditory) or the vibratory frequency (tactile) was elevated. Participants did not know that it was always two stimulation-blocks per run which contained such outlier-stimuli. Participants had to press a button with their left index finger when detecting an outlier. The amount of outlier-blocks was fixed, equally distributed across conditions, and randomized in temporal position across the seven runs. Taken together, each run comprised three blocks for each of the four condition plus two outlier blocks.

MRI data was acquired in 6 runs of 8.5 min and a structural scan on a 3 T TIM Trio (Siemens) at the Center for Cognitive Neuroscience Berlin (CCNB) of the Freie Universtät, Berlin. 255 functional images were acquired per run (T2*-weighted gradient-echo EPI: 37 contiguous slices; ascending order; 20% gap; whole brain; TR = 2000 ms; TE = 30 ms; 3 × 3 × 3 mm³ voxel; flip angle = 90°; 64 × 64 matrix) and structural MRI data (T1-weighted MPRAGE: 176 sagittal slices, TR = 1900, TE = 2.52 ms; 1 × 1 × 1 mm³ voxel).

### Univariate fMRI data analysis

All fMRI data processing was performed with SPM8 (Wellcome Trust Centre for Neuroimaging, Institute for Neurology, University College London, London, UK). To minimize movement-induced image artifacts each data set was realigned to its mean image. After estimating the inter-subject alignment by matching tissue class images together, the warping parameters were used to transform each subject’s fMRI volumes into MNI space where the EPI images were re-interpolated to 2 × 2 × 2 mm^3^ voxel size. Images were spatially smoothed with an 8 mm FWHM Gaussian kernel.

To test for differences in activation levels between the PRE to the POST scan, we computed classical GLM analyses for both measuring time points. On the first-level, we modelled independent regressors for the four experimental conditions (CON-Tactile, CON-Auditory, DIS-Tactile, DIS-Auditory) and computed contrasts between CON and DIS for auditory and tactile conditions. Consequently, we implemented a repeated measures ANOVA with the flexible factorial design option of SPM and contrasted PRE versus POST contrasts. Results were assessed at p < 0.05 family wise error (FWE) corrected for whole brain analyses, and at p < 0.001 uncorrected within anatomical masks of SI, SII, Hippocampus and AI.

### Connectivity analysis

To identify seed-regions for the connectivity analysis we used the results of the given GLM analysis. Task-dependent connectivity modulations were assessed with the seed-based connectivity measure of psychophysiological interaction (PPI) analysis as implemented in SPM8^23^. We focused our analysis on the a priori motivated seed-regions left primary somatosensory cortex (SI) and bilateral primary auditory cortices (AI). We defined 2 mm spheres around group-level peak voxels in the GLM main-effects of Tactile > Auditory and Auditory > Tactile, within anatomically defined regions using the Anatomy toolbox^25,26^. Spheres were defined around the coordinates as follows: left SI: x = −58, y = −18, z = 42; left AI: x = −44, y = −26, z = 8; right AI: x = 46, y = −28, z = 10. PPI analysis with seed-regions in SII were not conducted, due to the spatial proximity of auditory activation clusters with SII clusters that makes it problematic to identify a representative SII seed region (See Supplementary Figure S1). Note that the hippocampus was not used as a seed-region, as it did not show activation differences in the univariate analysis. Following the PPI approach, for each seed region the first Eigenvariate of the BOLD time-series was extracted. Next, the deconvolved time-series were multiplied by the psychological variables (CON-Auditory > DIS-Auditory contrast, expressed as onset weights [1–1]), and reconvolved with the HRF to obtain the PPI interaction-terms^23^. On the single-subject level our multi-run PPI design contained sets of three regressors per run: interaction-term, time-series and psychological factor^23^.

Each subject’s estimated PPI interaction term parameters were forwarded to a second-level ANOVA design (implemented as flexible factorial design in SPM). Herein we assessed the overlap of changes from the PRE to the POST scans for left and right AI with a conjunction analysis of the results from left and right AI seed regions (Conjunction against Global-Null hypothesis^27^). All reported coordinates correspond to MNI space. Results were assessed with a significance threshold of p < 0.001 uncorrected, within the *a priori* defined network of interest, constituted of bilateral primary (SI) and secondary somatosensory (SII) cortices, as well as bilateral hippocampi. The SPM anatomy toolbox was used to establish cytoarchitectonical references^26^. The identified clusters are displayed without masking to demonstrate their anatomical specificity as clearly delimited clusters.

## Results

### Behavioral results

Improvements in discrimination ability of tactile stimuli after the training phase are reported elsewhere^13^. In short, subjects significantly improved their discrimination ability of tactile stimuli from the CON condition in comparison to the DIS condition. During the fMRI session, participants had to perform an outlier detection task. Performance of PRE: 91.7 ± 7.7% and POST: 92.8 ± 12.9% (mean ± SD) detection accuracy indicates that participants kept their attention focused on the stimulus perception throughout the fMRI data acquisition.

### Univariate differences in activation levels

To test the hypothesis that the interaction of auditory language related processing and somatosensory perceptual regions induces cross-modal activation, we tested for activation increases from the PRE to the POST scans in the contrasts of CON and DIS conditions. None of the tested contrasts revealed significant differences when testing assumption free in a whole brain analysis on p < 0.05 FWE corrected. Also, testing with a more liberal threshold of p < 0.001 uncorrected within the depicted regions of interest did not reveal any voxels to show significant activation increases.

### Changes in coupling of auditory and somatosensory cortices

**Figure 2:**
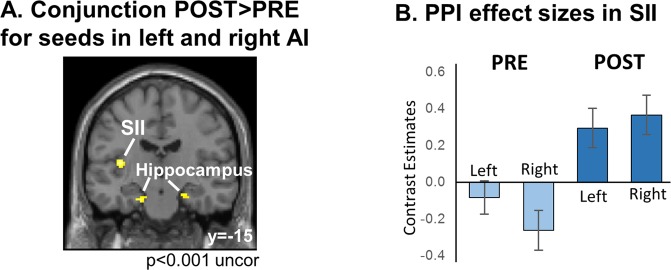
Changes in coupling between auditory and somatosensory cortices. The PPI approach allows quantifying task-modulated functional connectivity. Here we tested for coupling of the auditory cortex (seed-regions) during the presentation of pseudowords. More specifically, we assessed the coupling strength that is modulated by the difference between the CON and DIS condition, pre and post a one week training phase (see Fig. 1). (**A**) Conjunction analysis of PPI results from left and right primary auditory cortex (A1): Task modulated connectivity was tested for the contrast POST > PRE, identifying regions in the conjunction analysis that displayed an increase in coupling. As this PPI connectivity increase is specific for the task modulation CON versus DIS (see methods), it is specific to the consistent labelling of the stimuli through the training. (**B**) Effect sizes of the PPI analysis expressed as contrast estimates from the second-level ANOVA design: The reported effect demonstrates consistency in the analyses from left and right auditory cortex.

To test if the training has caused changes in the coupling strength between auditory and somatosensory regions we used the PPI approach^23^. We tested for an increase in PPI connectivity (modulated by the contrast CON versus DIS) from left SI and bilateral AI. The PPI analysis with seed-region in SI did not reveal increased coupling of SI to other brain regions when testing assumption free in a whole brain analysis on p < 0.05 FWE corrected. Additionally testing with a more liberal threshold of p < 0.001 uncorrected within the depicted regions of interest did not reveal any voxels to show significant activation increases. As PPI analyses are designed to assess connectivity changes for individual seed-regions, we conducted two independent analyses for the left and the right AI. In a second-level ANOVA design we then assessed the conjunction of both of these analyses within the given network of interest. While this analysis did not reveal changes in connectivity to primary somatosensory cortices, it did reveal bilateral hippocampi and a cluster in the left secondary somatosensory cortex. Please note that during training, tactile stimuli were presented to the right middle finger, leading to a predominant left hemispheric processing. The cluster in the right hippocampus was of 51 voxels size (peak: x = 22, y = −20, z = −20; z-score = 4.15), in the left hippocampus of 57 voxels (peak: x = −26, y = −12, z = −26; z-score = 4.16), and the left SII cluster span 20 voxels (peak: x = −38, y = −16, z = 10; z-score = 3.80) at p < 0.001 uncorrected (Fig. 2A). To show the consistency of this increase in task-modulated coupling from bilateral AI to the left SII, we plotted PPI effect sizes as contrast estimates for the PRE and POST scans in Fig. 2B.

## Discussion

In the present study, we used fMRI to investigate emerging neuronal networks underlying the concordant pairing of pseudowords with tactile stimuli before and after one week of intensive implicit training. A previous behavioural study demonstrated that the given implicit association training increased participants’ discrimination ability for tactile stimuli only after concordant pairing, in comparison to discordant pairing, with pseudowords. During fMRI scanning, participants were presented with unimodal presentation of either pseudowords or tactile stimuli. We did not find any training induced changes in activation levels related to the presentation of these stimuli. PPI analyses to test for changes in connectivity between CON and DIS conditions revealed consistent increases in coupling across bilateral primary auditory cortices and the left secondary somatosensory cortex for the presentation of the CON versus DIS pseudowords. Additionally, we observed increased coupling to bilateral hippocampi, which are well known for their contributions to association learning. The observed connectivity changes constitute evidence for an efficient language-to-sensory coupling of word form representations to sensory input representations, activating auditory-to-sensory loops. These brain mechanisms of functional coupling provide a physiological correlate of the behavioural improvement of tactile discrimination, which we previously documented for co-presented but task-irrelevant acoustic language stimuli.

Our results support Hebbian learning as a basic principle underlying the causal effects of auditory input, namely language-like spoken stimuli, on perception. By including a control condition with random labelling and equal exposure, we were able to demonstrate that it is the correlated pairing of acoustic-verbal labels with percepts that drives an increase in coupling between auditory and somatosensory cortices. On the contrary, exposure alone and uncorrelated discordant pairing of multiple percepts to multiple labels did not result in such a coupling increase. We used a paradigm with within-subject manipulation, eliminating any confounds that may arise when using between-group comparisons which were frequently used for testing putative effects of pre-established vocabulary differences. We avoided the use of meaningful words from natural languages, or the use of pre-established categories of objects, and the matched stimulus sets were fully counterbalanced across conditions. Using an implicit learning paradigm with unfamiliar stimuli and within-subject manipulation, where all participants were exposed to both the critical and control condition, rules out any potential confounding effects of the stimulus material, perceptual experience or pre-existing knowledge. Future studies need to address the important and still open question whether the observed interaction effects critically depend on the verbal linguistic nature of the stimuli and their acoustic nature, or could, in principle, be achieved with stimuli of any modality and complexity, regardless of whether they resemble words or not. This research must test, for example, whether similar effects can be found if tactile stimuli were paired with non-language stimuli such as meaningless simple sounds or elementary visual shapes and written words or manual gestures. Dependent on the results, the observed effect would need to be re-interpreted in terms of general mechanisms of multimodal association or specifically linguistic ones. Furthermore, future research needs to address which specific stimulus features are required for causing the observed *language perception causality* effect in behavior and the related modulation of low level interactions between sensory cortices documented in this present work.

The given study design controlled for changes that could be ascribed to fast adaptation during the measurement, as these would have been equally present within both the PRE as in the POST scanning sessions. This aspect of the design allows rendering of the given connectivity changes specific to the associations formed over the learning period and tapping into long-term effects rather than memory traces with rapid dynamics^28^.

### Activation Differences versus Changes in Connectivity

Previous studies have shown that certain words related to perception, such as olfaction, will activate the respective modality-preferential perceptual areas of the brain^29,30^. Univariate analysis in the given data set did not reveal any significant activation differences for tactile stimuli nor for pseudowords specific to the training throughout the brain. One reason for this may be that the activation difference for newly arising cell assemblies between language processing areas and somatosensory areas might be too small to be detectable with the sensitivity of the BOLD contrast, given that only a small vocabulary of frequently repeated labels were used in this study. In contrast, changes in coupling between brain regions can arise without major changes in activation levels, via synchronization of regions which retain the same overall mean activation levels.

Testing for changes in PPI connectivity of a seed region in the left primary somatosensory cortex during tactile stimulation did not reveal any learning induced changes from the PRE to the POST scanning session. This might indicate that the causal effect of language on perception is mainly mediated by the AI to SII coupling. One should, however, note that primary somatosensory cortex is also assumed to be more susceptible to subject-specific variability, which makes it problematic to identify an appropriate seed region for the given PPI seed-based analysis. Finding generally weaker activation in secondary somatosensory cortices than in primary auditory cortices did not allow us to properly test for changes in coupling of SII to other regions and needs to be addressed in future studies with adapted study designs. Note, however, that the seed region effect from the auditory to the somatosensory domain fits the causal effect of redundant verbal label presentation on tactile discrimination shown by our previous work^13^. Therefore, it may provide a brain mechanism for the facilitating effect of multimodal integration of the spoken pseudoword stimuli with information in early somatosensory regions.

Our identification of SII as a major site of learning adaptations within the somatosensory processing stream is in line with results from monkey research. The role of SII in the processing of vibrotactile stimuli has previously been studied mainly in the context of tactile stimulus perception, working memory, and decision making^31^. A series of studies by Romo and colleagues used electrophysiological recordings to test for correlates of stimulus discrimination and found firing patterns in SII related to perceptual as well as stimulus discrimination related processes^32^. Murray and Mishkin (1984) demonstrated that discrimination learning in rhesus monkey is most strongly disturbed after SII lesioning in comparison to other lesions within the somatosensory system. SII also demonstrates massive plastic changes after deafferentation, which is indicative of its high neuroplasticity, as required for any type of learning^33,34^.

It has previously been demonstrated that associative learning of word meanings engages limbic structures, in particular the hippocampus^35^. This learning may only be temporarily mediated during the first days/weeks of learning before transfer of memory representations to neocortex^36^, and activation in hippocampal regions would also be expected in the hereby investigated network. Indeed, we found this connection with bilateral hippocampi soon after learning.

### Association learning in language and Hebbian learning in the brain

Hebbian learning has been indicated to be the foundation of the emergence of various types of language related and multisensory integration processing. It is assumed to be the mechanism underlying learning-driven emergence of distributed neuronal circuits and their role in the process of semantic grounding, or embodiment, by which labels are related to perceptual or action-related information^37^. In contrast to complex semantic circuits, we here used the frequent co-occurrence of a tactile percept with a specific verbal label in the concordant condition, which would lead to co-activation of the neuronal circuits underpinning the tactile percept and that of the auditory percept located respectively in somatosensory and auditory areas. Even though exposure happened for several hours over the course of a week, there were no discrimination improvements for tactile stimuli from the control condition, only for those stimuli paired consistently with labels. We opted to train for one week, as our stimuli were very difficult to discriminate and it has been postulated that effects of language on perception are only present when the perceptual stimuli are difficult to discriminate^6,38^. While learning to associate word-referent pairs has been shown to happen very quickly when the referents are easily distinguishable^39^, learning to differentiate fine-grained perceptual representations may take longer^40^. Using PPI analysis, we showed that the activation dynamics between sensorimotor and auditory cortex becomes correlated after repeated, consistent pairing of individual labels with individual percepts, which is consistent with the emergence of distributed cell-assembly circuits as a result of Hebbian learning^15^.

### Conclusions

This study demonstrates that the use of verbal labels correlated with tactile stimuli can lead to the emergence of auditory-somatosensory neuronal networks, thus providing a putative physiological correlate of facilitative effects of language on perception. In a previous study, concordant verbal labels were able to influence the discrimination of fine-grained tactile-patterned stimuli after 1 week of co-presentation. Here, we report a plausible neuronal correlate of these behavioural findings. We observed an increase in coupling specific to concordantly labelled stimuli from pre to post training. This coupling was between bilateral primary auditory cortices and the left secondary somatosensory cortex, as well as bilateral hippocampus. These results provide a mechanistic explanation for the causal effects of language on perception as formulated by the *language perception causality* statement. Such a link may provide an explanation for why having specific words or labels for specific perceptual representations, such as colors, may alter the way that we perceive our environment.

## Supplementary information

Supplementary Material

## Data Availability

The dataset generated and analysed during the current study is available from the corresponding author on reasonable request.
