# Supplementary material for: Neuronal correlates of label facilitated tactile perception
Source: Sci Rep. 2019 Feb 7;9:1606. doi: 10.1038/s41598-018-37877-w (PMC6367477; doi:10.1038/s41598-018-37877-w)
Supplement: Supplementary file 1 — Supplementary Material [file 41598_2018_37877_MOESM1_ESM.pdf]

Supplementary Information

Neuronal correlates of label facilitated tactile perception

Timo Torsten Schmidt*, Tally McCormick Miller*, Felix Blankenburg, Friedemann Pulvermüller

* These authors contributed equally to this work

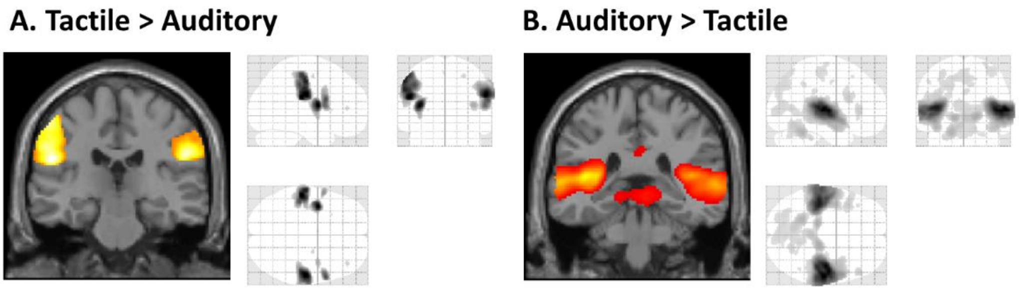

Supplementary Figure 1: Main effects of **A.** Tactile and **B.** Auditory stimulation across PRE and POST scan. Results displayed at $p<0.05$ FWE corrected.
